# Effect of Cardioprotection on the Right Ventricular Function in Breast Cancer Patients Receiving Potentially Cardiotoxic Therapy: A SAFE Trial Substudy

**DOI:** 10.1111/echo.70291

**Published:** 2025-09-18

**Authors:** Maria Riccarda Del Bene, Icro Meattini, Giuseppe Pilato, Carlotta Becherini, Francesca Martella, Viola Salvestrini, Livia Marrazzo, Calogero Saieva, Iacopo Olivotto, Giuseppe Barletta, Lorenzo Livi

**Affiliations:** ^1^ Diagnostic Cardiology CardioThoracic and Vascular Department Careggi University Hospital Florence Italy; ^2^ Department of Experimental and Clinical Biomedical Sciences “M. Serio” University of Florence Florence Italy; ^3^ Radiation Oncology and Breast Unit Careggi University Hospital Florence Italy; ^4^ Breast and Medical Oncology Units Oncology Department Azienda USL Toscana Centro Florence Italy; ^5^ Medical Physics Unit Oncology Department Careggi University Hospital Florence Italy; ^6^ Cancer Risk Factors and Lifestyle Epidemiology Unit Institute For Cancer Research Prevention and Clinical Network (ISPRO) Florence Italy; ^7^ Department of Experimental and Clinical Medicine University of Florence Florence Italy

**Keywords:** 3D echocardiography, anthracycline, breast cancer, chemotherapy‐induced cardiotoxicity, neurohormonal inhibitors, right ventricular function

## Abstract

**Purpose:**

To evaluate the effects of cardioprotective therapy (CPT) with neurohormonal inhibitors on cancer therapeutics–related cardiac dysfunction (CTRCD) in breast cancer patients, focusing on right ventricular (RV) function.

**Methods:**

This is a secondary analysis of SAFE study, a randomized, phase 3, double‐blind, placebo‐controlled, four‐arm trial, in which the effects of short‐term CPT (bisoprolol, ramipril, or both) compared to placebo (P‐arm) on subclinical CTRCD were evaluated in 222 women without cardiac risk factors who received intensive anthracycline‐based chemotherapy (median isoequivalent doxorubicin dose 288 mg/m^2^). Among them, 35% received trastuzumab, 98% taxanes, 22% underwent neoadjuvant therapy, 78% adjuvant therapy, and 56% had postoperative radiotherapy. CPT started with chemotherapy and continued for 1 year, or until the completion of trastuzumab therapy. All the patients underwent cardiac surveillance at baseline and 3, 6, 12, and 24 months. Left ventricular CTRCD was assessed following the 2022 ESC guidelines. RV function was evaluated according to established recommendations. RV CTRCD was defined as a greater than 10% reduction in RV fractional area change (FAC).

**Results:**

At 24 months, LV CTRCD was observed in 42.9% of P‐arm and 3.1% of the CPT arms (*p* < 0.001). Compared to the CPT arms, there was a significant reduction in RV FAC (−10.5%), S’‐wave velocity (−12.2%), and tricuspid annular plane systolic excursion (−9.6%) in the P‐arm. Additionally, the RV diameter increased by 7% in the P‐arm. RV CTRCD was found in 49.2% of the P‐arm and 22% of the CPT arms (*p* < 0.001).

**Conclusion:**

Short‐term neurohormonal cardioprotection was effective in reducing RV CTRCD.

## Graphical abstract

Short‐term neurohormonal cardioprotection reduced both left and right ventricular deterioration in breast cancer patients undergoing anthracycline‐based chemotherapy. Administration of bisoprolol, ramipril, or a combination of both mitigated the decline in right ventricular function during chemotherapy compared to placebo, facilitating recovery at 2 years with minimal loss of function.

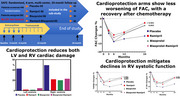

**Abbreviations**
ACE: angiotensin‐converting enzyme
ARBs: angiotensin‐II receptor blockers
CPT: cardioprotective therapy
CTRCD
: cancer therapy‐related cardiac dysfunction
ECG: electrocardiogram
EOS: end of study
EOT: end of treatment
FAC: fractional area change
GLS: global linear strain
IVCD: inferior vena cava diameter
LV: left ventricle
LVEF: left ventricular ejection fraction
P: placebo
RAV: right atrial volume
RV: right ventricle
S'RV: RV pulsed tissue Doppler systolic wave velocity
SPAP: systolic pulmonary artery pressure
TAPSE: tricuspid annular plane systolic excursion

## 1 Introduction

Cancer therapy–related cardiac dysfunction (CTRCD) is a condition that mainly affects the left ventricle. Over the years, various criteria have been adopted to diagnose CTRCD, focusing on the left ventricular ejection fraction (LVEF) [1]. The most recent guidelines state that CTRCD should be diagnosed when there is a decrease in the LVEF of more than 10 percentage points, resulting in a value of less than 50%. Probable cardiotoxicity is reflected by a decrease in the LVEF of more than 10 percentage points, resulting in a value of 50% or more and a decline in global longitudinal strain (GLS) of more than 15% from the baseline. Possible CTRCD by echocardiography is recognized when the LVEF decreases by less than 10 percentage points, resulting in a value of less than 50%, or when there is a relative percentage reduction in GLS of more than 15% [2, 3]. Three‐dimensional LVEF is strongly recommended as it is more reliable [4].

There is growing evidence that abnormalities in the structure and function of the right ventricle may be significant in predicting the outcome of patients with CTRCD [5, 6]. However, the traditional definition of CTRCD has not included the assessment of right ventricular (RV) abnormalities. Studies on breast cancer patients have reported changes in various RV parameters at different degrees [7]. Still, no trials have yet been conducted to evaluate the effects of cardioprotective therapy (CPT) based on neurohormonal inhibitors on the right ventricle. Only one study has evaluated the effect of dexrazoxane on RV myocardial deformation parameters in breast cancer patients treated with doxorubicin, showing a modest improvement in treated patients [8].

The SAFE study is a randomized, placebo‐controlled study, whose final results have been published recently [9]. Its objective was to assess the effect of short‐term CPT with neurohormonal inhibitors on subclinical CTRCD in women with nonmetastatic breast cancer, who had a low cardiovascular risk profile and were planned to receive intense up‐to‐date anthracycline‐based chemotherapy.

In this study, we present the analysis of the SAFE trial data relative to the right ventricle. The main objectives are as follows: (1) to assess how intensive breast cancer therapy affects RV function and the relation with LV CTRCD, and (2) the effect of cardioprotection during chemotherapy on RV function.

## 2 Materials and Methods

The SAFE trial was a phase 3, double‐blind, randomized, and placebo‐controlled study with a four‐arm design, conducted in three centers in Italy. Its purpose was to evaluate the effect of bisoprolol, ramipril, or a combination of both drugs compared to placebo on subclinical LV dysfunction assessed by 3D‐LVEF and speckle tracking (GLS) cardiac echocardiography in nonmetastatic breast cancer patients. The trial protocol was previously published [10].

The trial included women aged 18 or older with nonmetastatic, histology‐proven, invasive breast carcinoma. They were eligible for primary or postoperative systemic therapy using an anthracycline‐based regimen with or without trastuzumab. The exclusion criteria were patients who had received anthracycline treatment previously, were currently receiving angiotensin‐converting enzyme inhibitors/angiotensin‐II receptor blockers (ARBs) or beta‐blockers, had a baseline LVEF below 50%, had a prior diagnosis of solid tumors treated with systemic therapy, were unable to undergo evaluation of LV function by echocardiography, had symptoms of heart failure, had a prior diagnosis of cardiomyopathy, coronary artery disease, moderate‐to‐severe mitral or aortic disease, or were receiving pharmacological therapy for asthma, hypercholesterolemia, diabetes, and hypertension. All patients provided written informed consent.

The trial administered CPT for 1 year from the initiation of chemotherapy or until the end of trastuzumab therapy. Doses for all groups were uptitrated at 1‐week intervals, up to the target dose of bisoprolol (5 mg), ramipril (5 mg), and placebo, if tolerated. All patients underwent cardiac surveillance at baseline, 3 months, 6 months, 12 months (end of treatment—EOT), and 24 months (end of study—EOS) from enrollment. Compliance with the study drugs was generally good, with 91.4% of patients not requiring dose downtitration or discontinuation. To note, patients in the bisoprolol plus ramipril arm had a higher rate of dose downtitration or discontinuation (15%) and a higher incidence of hypotension (8.3%) and cough (5%) compared to the other treatment arms.

During each medical visit, the patient's medical history, electrocardiogram (ECG), clinical examination with specific attention to signs of heart failure, NYHA class, and Canadian Angina Grading Scale Score were recorded. N‐terminal pro‐B‐type natriuretic peptide (NT‐proBNP) was dosed at each scheduled time point. Transthoracic echocardiography was performed using a commercially available system (EPIQ, X5‐1 transducer, Philips Healthcare, Andover, Massachusetts), and all measurements were performed and reported following the American Society of Echocardiography (ASE) and the European Association of Cardiovascular Imaging (EACVI) recommendations [11]. Each measure was averaged over three cardiac cycles.

**FIGURE 1:**
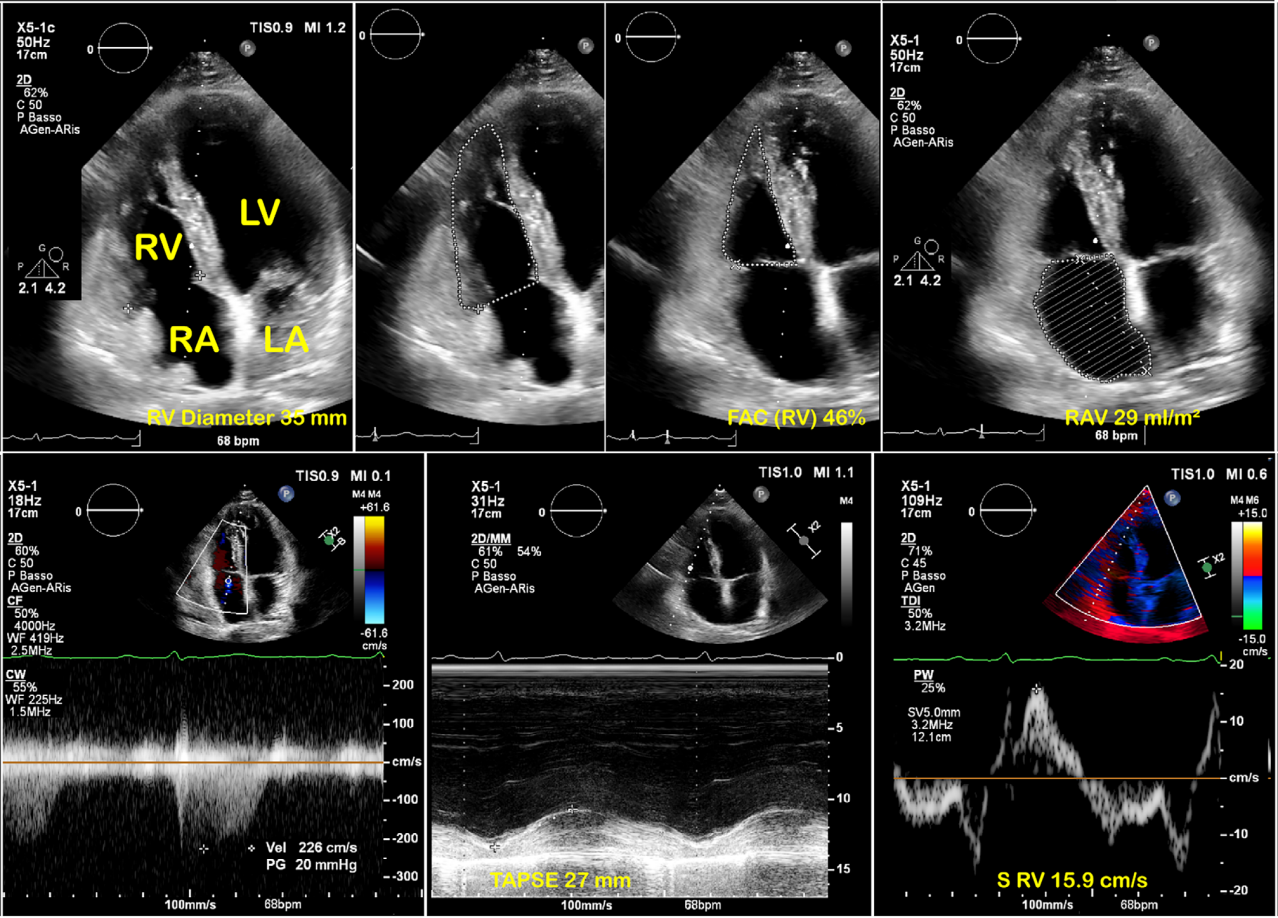
Representative two‐dimensional right‐oriented four‐chamber images to measure (from left to right) the right ventricular diameter, diastolic and systolic areas used to calculate the fractional area change (FAC), and the right atrium volume (RAV) (top panel). On the bottom panel, the continuous‐wave Doppler tricuspid flow velocity from which the tricuspid systolic pressure gradient is calculated, the tricuspid annular plane systolic excursion (TAPSE), and the systolic velocity of tricuspid annulus (S’) by tissue Doppler imaging.

In the focused RV apical four‐chamber view at end‐diastole, the RV basal diameters were measured. RV end‐diastolic and end‐systolic areas were measured to calculate RV fractional area change (FAC). Tricuspid annular plane systolic excursion (TAPSE) by the M‐mode approach in the apical four‐chamber view and RV pulsed tissue Doppler systolic wave velocity (S'RV) were also measured [12]. Right atrial volume (RAV) was calculated by single‐plane disk summation techniques in a dedicated apical four‐chamber view [11]. Systolic pulmonary artery pressure (SPAP) was estimated using the continuous wave Doppler of the tricuspid regurgitation jet. Representative imaging of the RV measures is shown in Figure 1. In the subcostal view with the patient in the supine position at 1.0–2.0 cm from the junction of the right atrium, the inferior vena cava diameter (IVCD) was measured. The right atrial pressure was estimated based on IVCD and its collapse after sniffing.

To obtain LV GLS measurements, after optimizing image quality, maximizing frame rate, and minimizing foreshortening, the three standard apical views were acquired during a breath hold. Full‐volume four to six‐cycle gated acquisition breath‐hold images of the left ventricle were acquired for Q‐lab analysis to obtain 3D‐LVEF [13]. Q‐lab version in this study was 15.5.

All echocardiography data, including the original Digital Imaging and Communications in Medicine (DICOM) images, were stored. All scans were centralized and read jointly by two experienced board‐certified echocardiographers (G.B. and M.R.D.B) blinded to all clinical characteristics. Systemic arterial pressure was measured simultaneously with echo measurements through an arm‐cuff sphygmomanometer. ECG was performed using ELI 200 Mortara electrocardiograph. The heart rate and QTc measurement, according to Fridericia correction, were recorded.

### 2.1 Statistical Analysis

To compare the individual characteristics of patients allocated to different arms at baseline, the chi‐squared test or Fisher's exact test was used for categorical variables, and ANOVA and Bonferroni *t*‐test were employed for continuous variables. ANCOVA analysis was conducted by comparing echocardiography imaging measures and the hemodynamic effects of the study drug in the placebo group with each drug arm using *t*‐tests with Bonferroni correction. Logistic univariate and multivariate regression analysis was used to determine predictors of RV subclinical cardiotoxicity at EOS. Subclinical cardiotoxicity was indicated by a ≥ 10% worsening FAC at EOS, and clinical data were used as the independent variable. The statistical analyses were conducted using IBM SPSS version 26.0 (Armonk, NY).

## 3 Results

This secondary analysis was performed on the 222 patients who completed all the planned cardiological assessments from baseline to EOS. The baseline characteristics, including demographics, cardiovascular risk profile, and tumor anatomical and immunological profile, were similar across all groups (as shown in Table 1). All patients were given at least one cycle of anthracyclines (with a range of 1–6 cycles), and 97% of them were given at least three cycles. The median isoequivalent doxorubicin dose per patient was 288 mg/m^2^. Additionally, 217 patients received taxane, and 77 were administered adjuvant trastuzumab. Forty‐nine patients received neoadjuvant chemotherapy, 154 were given adjuvant endocrine therapy, and 124 had postoperative radiation therapy.

**TABLE 1:** Baseline characteristics of the study population.

Characteristic	Placebo *n* = 63	Ramipril *n* = 45	Bisoprolol *n* = 54	Bisoprolol—Ramipril *n* = 60	Total *n* = 222
Median age at diagnosis (years)	48.2	47.8	47.2	50.5	48.5
Height (cm)	162	166	164	163	164
Weight (kg)	62	65	64	62	63
Arterial pressure (Systolic/Diastolic) (mmHg)	122/74	124/76	121/74	124/74	123/74
Heart rate (bpm)	73	74	72	73	73
Hb (g/mL)	13.1	13.2	13	13.4	13.2
NT‐proBNP (pg/mL)	53	55	54	50	53
Menopausal status, no. (%)					
Premenopausal	39 (61.9)	29 (64.4)	39 (72.2)	32 (53.3)	139 (62.6)
Postmenopausal	24 (38.1)	16 (35.6)	15 (27.8)	28 (46.7)	83 (37.4)
Smoking habit, no. (%)					
No	48 (76.2)	32 (71.1)	37 (68.5)	43 (71.7)	160 (72.1)
Former/current	15 (23.8)	13 (28.9)	17 (31.5)	17 (28.3)	62 (27.9)
Body Mass Index					
<25	44 (69.8)	32 (71.1)	37 (68.5)	45 (75.0)	158 (71.2)
≥25	19 (30.2)	13 (28.9)	17 (31.5)	15 (25.0)	64 (28.8)
Breast surgery, no. (%)					
Breast conserving surgery	32 (50.8)	22 (48.9)	27 (50)	28 (46.7)	110 (49.5)
Mastectomy	31 (49.2)	23 (51.1)	27 (50)	32 (53.3)	112 (50.5)
Breast side, no. (%)					
Left	30 (47.6)	24 (53.3)	24 (44.4)	31 (51.7)	109 (49.1)
Right	33 (52.4)	21 (46.7)	30 (55.6)	29 (48.3)	113 (50.9)
Final surgical margins, no. (%)					
Negative	57 (90.5)	44 (97.8)	47 (87)	52 (86.7)	200 (90.1)
Positive	6 (9.5)	1 (2.2)	7 (13)	8 (13.3)	22 (9.9)
Axillary surgery, no. (%)					
Sentinel‐node biopsy	21 (33.3)	22 (48.9)	27 (50)	22 (36.7)	92 (41.4)
Axillary lymph nodes dissection	42 (66.7)	23 (51.1)	27 (50)	38 (63.3)	130 (58.6)
HER2 status, no. (%)					
Negative	42 (66.7)	28 (62.2)	32 (59.3)	40 (66.7)	142 (64)
Positive	21 (33.3)	17 (37.8)	22 (40.7)	20 (33.3)	80 (36)
Hormonal receptors status, no. (%)					
Positive	46 (73)	37 (82.2)	40 (74.1)	43 (71.7)	166 (74.8)
Negative	17 (27)	8 (17.8)	14 (25.9)	17 (28.3)	56 (25.2)
Postoperative radiation therapy, no. (%)	36 (57.1)	24 (53.3)	32 (59.3)	32 (53.3)	124 (55.9)
Left‐sided breast irradiation, no. (%)^a^	17 (47.2)	15 (33.3)	13 (40.6)	19 (59.4)	64 (51.6)
Radiation therapy volume, no. (%)^a^					
Whole breast irradiation	26 (72.2)	17 (70.8)	23 (71.9)	22 (68.7)	88 (71)
Postmastectomy radiation therapy	10 (27.8)	7 (29.2)	9 (28.1)	10 (31.3)	36 (29)
Regional nodal irradiation, no. (%)^a^	13 (36.1)	8 (33.3)	9 (28.1)	12 (37.5)	42 (33.9)
Tumor bed boost irradiation, no. (%)^a^	26 (72.2)	17 (70.8)	23 (71.9)	22 (68.7)	88 (71)
Chemotherapy intent, no. (%)					
Neoadjuvant	14 (22.2)	11 (24.4)	8 (14.8)	16 (26.7)	49 (22.1)
Adjuvant	49 (77.8)	34 (75.6)	46 (85.2)	44 (73.3)	173 (77.9)
Chemotherapy regimen, no. (%)					
Trastuzumab‐containing	20 (31.7)	16 (35.6)	21 (38.9)	20 (33.3)	77 (34.7)
Taxane‐containing	62 (98.4)	43 (95.6)	53 (98.1)	59 (98.3)	217 (97.7)
Adjuvant endocrine therapy, no. (%)	42 (66.7)	34 (75.6)	37 (68.5)	41 (68.3)	154 (69.4)

^a^ Percentages refer to patients treated with postoperative radiation therapy. Abbreviation: HER2: human epidermal growth factor receptor 2.

The overall LV CTRCD evaluated according to the 2022 ESC guidelines was 14.4%. CRTCD development was statistically different in the arms: 42.9% in the Placebo arm, 1.9%, 2.2%, and 5.0% in the Bisoprolol, Ramipril, and Ramipril–Bisoprolol arms, respectively (chi‐squared 57.954, *p* < 0.001).

**FIGURE 2:**
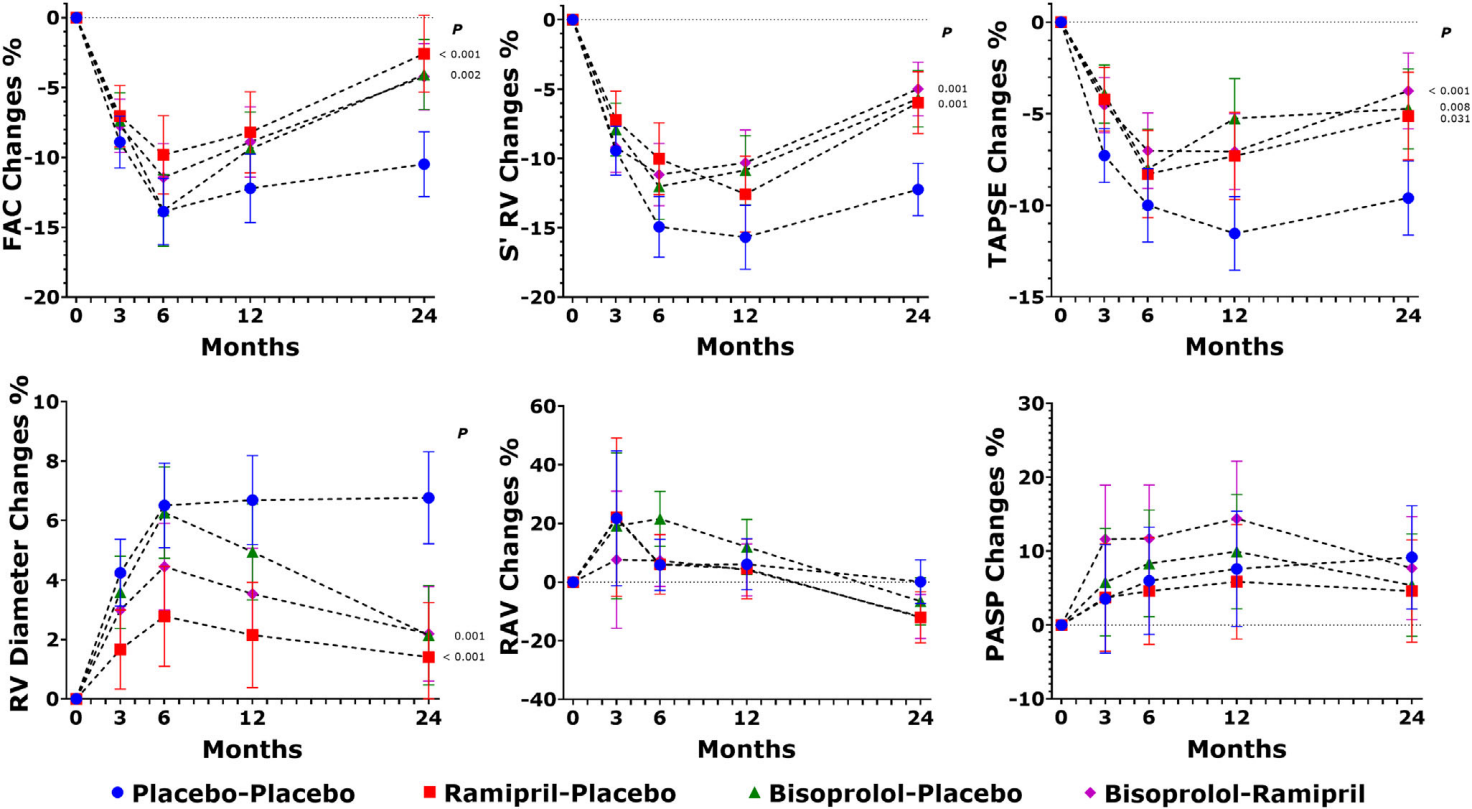
Trajectories of the percent changes over time of the right ventricular parameters. Color‐coded symbols identify the study arm as indicated at the bottom. Abbreviations: FAC: fractional area change; PASP: pulmonary artery systolic pressure; RAV: right atrium volume; RV: right ventricular; S'RV: pulsed tissue Doppler systolic wave velocity; TAPSE: tricuspid annular plane systolic excursion.

Table 2 reports the echocardiography measures relative to the right ventricle, pulmonary artery pressure, systemic blood pressure, and heart rate recorded at each surveillance visit by arm. The percent changes over time of the RV parameters are reported in Figure 2.

**TABLE 2:** Right ventricular echocardiographic measures, NT‐proBNP, arterial pressure, and heart rate.

Arm	EOS, No.	Baseline unadjusted	Adjusted			
			3‐month	6‐month	12‐month	24‐month
**SAP, mmHg**						
Placebo	63	122.4 ± 1.3 (119.7–125.1)	119.3 ± 1.3 (116.8–121.8)	121.4 ± 1.3 (118.9–123.9)	122.1 ± 1.3 (119.5–124.7)	121.9 ± 1.2 (119.5–124.2
Ramipril	45	123.9 ± 1.5 (120.8–127.0)	116.3 ± 1.5 (113.3–119.3)	116.8 ± 1.5 (113.9–119.8)	118.5 ± 1.6 (119.5–124.7)	120.8 ± 1.4 (118.0–123.6
Bisoprolol	54	120.6 ± 1.4 (117.8–123.5)	116.7 ± 1.4 (113.9–119.4)	118.9 ± 1.4 (116.2–121.6)	120.2 ± 1.5 (119.5–124.7)	122.4 ± 1.3 (119.8–124.9
Bisoprolol–Ramipril	60	124.5 ± 1.5 (121.5–127.4)	113.0 ± 1.3 (110.4–115.6)^‡^	111.0 ± 1.3 (108.5–113.6)^§^	113.8 ± 1.4 (119.5–124.7)^§^	120.0 ± 1.2 (117.6–122.5)
*All*	222	122.8 ± 0.7 (121.4–124.3)				
**DAP, mmHg**						
Placebo	63	74.3 ± 1.0 (72.3–76.4)	73.2 ± 0.9 (71.4–74.9)	74.7 ± 0.9 (72.9–76.4)	74.0 ± 0.9 (72.1–75.8)	74.5 ± 0.8 (72.9–76.1)
Ramipril	45	75.6 ± 1.3 (73.0–78.2)	70.4 ± 1.0 (68.4–72.5)	72.1 ± 1.0 (70.0–74.1)	72.8 ± 1.1 (70.7–75.0)	75.1 ± 1.0 (73.2–77.1)
Bisoprolol	54	73.7 ± 1.2 (71.3–76.1)	71.3 ± 1.0 (69.4–73.2)	71.8 ± 1.0 (69.9–73.7)	71.8 ± 1.0 (69.8–73.8)	75.9 ± 0.9 (74.2–77.7)
Bisoprolol–Ramipril	60	74.2 ± 1.2 (71.9–76.5)	68.3 ± 0.9 (66.5–70.1)^‡^	69.0 ± 0.9 (67.2–70.8)^§^	69.7 ± 1.0 (67.8–71.6)^†^	75.5 ± 0.8 (73.9–77.2)
*All*	222	74.4 ± 0.6 (73.3–75.5)				
**HR, bpm**						
Placebo	63	72.7 ± 1.3 (70.0–75.3)	78.8 ± 1.2 (76.4–81.2)	79.5 ± 1.4 (76.8–82.3)	71.8 ± 1.2 (69.4–74.2)	71.3 ± 1.3 (68.8–73.8)
Ramipril	45	74.6 ± 1.5 (71.6–77.6)	79.3 ± 1.4 (76.5–82.2)	77.7 ± 1.7 (74.5–81.0)	69.4 ± 1.4 (66.6–72.3)	71.9 ± 1.5 (69.0–74.9)
Bisoprolol	54	72.2 ± 1.3 (69.6–74.9)	65.6 ± 1.3 (63.0–68.2)^§^	67.5 ± 1.5 (64.5–70.4)^§^	61.2 ± 1.3 (58.6–63.7)^§^	68.5 ± 1.4 (65.8–71.1)
Bisoprolol–Ramipril	60	73.0 ± 1.6 (69.7–76.3)	67.7 ± 1.2 (65.3–70.2)^§^	68.9 ± 1.4 (66.0–71.7)^§^	64.3 ± 1.2 (61.8–66.7)^§^	71.6 ± 1.3 (69.0–74.1)
**RV Diameter, mm**						
Placebo	63	31.7 ± 0.4 (30.8–32.5)	33.3 ± 0.2 (32.9–33.6)	33.9 ± 0.2 (33.5–34.3)	34.0 ± 0.2 (33.6–34.4)	34.0 ± 0.2 (33.6–34.5)
Ramipril	45	32.7 ± 0.4 (32.0–33.5)	32.7 ± 0.2 (32.3–33.1)	33.1 ± 0.2 (32.6–33.6)	32.9 ± 0.3 (32.4–33.4)^†^	32.6 ± 0.3 (32.1–33.2)^‡^
Bisoprolol	54	31.9 ± 0.4 (31.1–32.7)	33.1 ± 0.2 (32.8–33.5)	34.0 ± 0.2 (33.5–34.4)	33.5 ± 0.2 (33.1–34.0)	32.7 ± 0.2 (32.2–33.2)^‡^
Bisoprolol–Ramipril	60	32.1 ± 0.3 (31.5–32.8)	33.0 ± 0.2 (32.7–33.3)	33.5 ± 0.2 (33.0–33.9)	33.2 ± 0.2 (32.7–33.6)^*^	32.7 ± 0.2 (32.2–33.2)^§^
*All*	222	32.1 ± 0.2 (31.7–32.4)				
**FAC, %**						
Placebo	63	48.1 ± 0.5 (47.0–49.2)	44.1 ± 0.5 (43.2–45.0)	41.6 ± 0.6 (40.5–42.7)	42.3 ± 0.6 (41.2–43.4)	43.2 ± 0.5 (42.1–44.2)
Ramipril	45	47.9 ± 0.6 (46.6–49.1)	44.9 ± 0.5 (43.9–46.0)	43.4 ± 0.7 (42.1–44.7)	44.1 ± 0.7 (42.8–45.4)	46.9 ± 0.6 (45.6–48.1)^§^
Bisoprolol	54	49.2 ± 0.6 (47.9–50.4)	45.1 ± 0.5 (44.1–46.0)	41.9 ± 0.6 (40.8–43.1)	44.1 ± 0.6 (42.9–45.3)	46.8 ± 0.6 (45.7–47.9)^§^
Bisoprolol–Ramipril	60	49.4 ± 0.6 (48.1–50.7)	45.0 ± 0.5 (44.1–46.0)	43.2 ± 0.6 (42.1–44.3)	44.5 ± 0.6 (43.4–45.7)^*^	46.7 ± 0.5 (45.7–47.8)^§^
*All*	222	48.7 ± 0.3 (48.1–49.3)				
**TAPSE, mm**						
Placebo	63	24.4 ± 0.3 (23.8–25.0)	22.5 ± 0.2 (22.2–22.9)	21.8 ± 0.2 (21.4–22.3)	21.4 ± 0.2 (21.0–21.9)	21.9 ± 0.2 (21.4–22.4)
Ramipril	45	23.9 ± 0.3 (23.4–24.4)	23.3 ± 0.2 (22.8–23.7)	22.2 ± 0.3 (21.6–22.7)	22.4 ± 0.3 (21.8–22.9)	22.9 ± 0.3 (22.3–23.5)
Bisoprolol	54	24.5 ± 0.3 (24.0–25.0)	23.4 ± 0.2 (23.0–23.8)^†^	22.4 ± 0.3 (21.9–22.9)	23.1 ± 0.3 (22.6–23.6)^§^	23.2 ± 0.3 (22.7–23.7)^‡^
Bisoprolol–Ramipril	60	24.4 ± 0.2 (23.9–24.9)	23.2 ± 0.2 (22.9–23.6)^*^	22.6 ± 0.2 (22.1–23.1)	22.6 ± 0.2 (22.1–23.1)^‡^	23.4 ± 0.2 (22.9–23.9)^§^
*All*	222	24.3 ± 0.1 (24.1–24.6)				
**S'RV, cm/s**						
Placebo	63	13.2 ± 0.2 (12.9–13.6)	11.7 ± 0.1 (11.5–11.9)	11.0 ± 0.1 (10.8–11.3)	10.9 ± 0.1 (10.7–11.2)	11.4 ± 0.1 (11.1–11.6)
Ramipril	45	12.8 ± 0.2 (12.5–13.2)	11.9 ± 0.1 (11.7–12.2)	11.6 ± 0.2 (11.2–11.9)	11.2 ± 0.2 (10.9–11.6)	12.1 ± 0.1 (11.8–12.4)^‡^
Bisoprolol	54	12.8 ± 0.2 (12.5–13.1)	11.9 ± 0.1 (11.6–12.1)	11.3 ± 0.1 (11.0–11.6)	11.4 ± 0.2 (11.1–11.8)	12.1 ± 0.1 (11.9–12.4)^§^
Bisoprolol–Ramipril	60	12.8 ± 0.2 (12.5–13.1)	11.7 ± 0.1 (11.5–11.9)	11.4 ± 0.1 (11.1–11.7)	11.5 ± 0.2 (11.2–11.8)^*^	12.2 ± 0.1 (12.0–12.5)^§^
*All*	222	12.9 ± 0.1 (12.7–13.1)				
**PASP, mmHg**						
Placebo	61	24.7 ± 0.7 (23.3–26.1)	22.7 ± 0.6 (21.6–23.9)	23.3 ± 0.5 (22.3–24.2)	23.7 ± 0.5 (22.6–24.8)	23.9 ± 0.5 (22.9–24.8)
Ramipril	45	24.6 ± 0.7 (23.2–26.0)	22.6 ± 0.7 (21.3–23.9)	22.5 ± 0.6 (21.3–23.6)	22.9 ± 0.6 (21.6–24.2)	23.0 ± 0.6 (21.9–24.1)
Bisoprolol	52	23.1 ± 0.7 (21.7–24.5)	24.0 ± 0.6 (22.8–25.2)	23.8 ± 0.5 (22.8–24.9)	24.6 ± 0.6 (23.4–25.7)	23.3 ± 0.5 (22.3–24.4)
Bisoprolol–Ramipril	58	22.9 ± 0.8 (21.3–24.4)	25.0 ± 0.6 (23.9–26.2)^*^	24.2 ± 0.5 (23.2–25.1)	24.4 ± 0.6 (23.3–25.5)	23.5 ± 0.5 (22.5–24.4)
*All*	216	23.8 ± 0.4 (23.1–24.5)				
**RAV, mL/m^2^**						
Placebo	63	19.3 ± 0.6 (18.1–20.5)	22.7 ± 2.1 (18.5–26.9)	19.7 ± 0.7 (18.4–21.0)	20.0 ± 0.7 (18.6–21.3)	18.7 ± 0.6 (17.5–19.8)
Ramipril	45	20.1 ± 0.7 (18.7–21.4)	23.4 ± 2.5 (18.3–28.4)	20.4 ± 0.8 (18.9–22.0)	20.0 ± 0.8 (18.4–21.6)	16.9 ± 0.7 (15.5–18.3)
Bisoprolol	54	18.9 ± 0.7 (17.5–20.4)	22.1 ± 2.3 (17.5–26.7)	22.5 ± 0.7 (21.0–23.9)^*^	20.5 ± 0.7 (19.0–21.9)	17.4 ± 0.6 (16.1–18.6)
Bisoprolol–Ramipril	60	19.6 ± 0.6 (18.4–20.8)	20.5 ± 2.2 (16.2–24.8)	20.3 ± 0.7 (18.9–21.6)	19.6 ± 0.7 (18.3–21.0)	16.6 ± 0.6 (15.5–17.8)
*All*	222	19.4 ± 0.3 (18.8–20.1)				
**NT‐proBNP, pg/mL**						
Placebo	63	54.5 ± 3.0 (48.5–60.4)	76.1 ± 5.0 (66.2–86.0)	84.8 ± 3.9 (77.1–92.5)	83.9 ± 3.7 (76.7–91.1)	90.5 ± 3.8 (83.0–98.0)
Ramipril	45	55.2 ± 3.0 (49.3–61.1)	68.4 ± 4.9 (58.8–78.0)	78.4 ± 3.8 (70.9–85.8)	75.0 ± 3.5 (68.1–82.0)	68.5 ± 3.7 (61.2–75.8)^§^
Bisoprolol	54	60.3 ± 3.0 (54.4–66.2)	65.8 ± 4.6 (56.7–74.8)	80.1 ± 3.6 (73.1–87.1)	78.3 ± 3.3 (71.8–84.9)	72.0 ± 3.5 (65.1–78.9)^‡^
Bisoprolol–Ramipril	60	57.6 ± 3.0 (51.6–63.5)	71.4 ± 4.8 (62.0–80.7)	82.2 ± 3.7 (75.0–89.5)	79.0 ± 3.5 (72.2–85.8)	74.4 ± 3.6 (67.3–81.5)^*^
*All*	222	56.9 ± 1.5 (53.9–59.8)				

*Note*: Data are reported as mean ± SE (95% CI). Statistical analysis: ANCOVA and *t*‐test with Bonferroni correction.

Abbreviations: DAP: diastolic arterial pressure; FAC: fractional area change; HR: heart rate; NT‐proBNP: N‐terminal pro‐B‐type natriuretic peptide; PAPS: pulmonary artery systolic pressure; RAV: right atrium systolic volume; RV: right ventricular; S’: pulsed tissue Doppler systolic wave velocity; SAP: systolic arterial pressure; TAPSE: tricuspid annular plane systolic excursion.

*p* value versus Placebo: ^*^ < 0.05; ^†^ < 0.01; ^‡^ < 0.005; ^§^ < 0.001.

In each arm, FAC decreased at 3 months (Placebo −8.9%, Ramipril −7.0%, Bisoprolol −7.4%, Bisoprolol–Ramipril −7.7%), further decreasing at 6 months (Placebo −13.9%, Ramipril −9.8%, Bisoprolol −13.8%, Bisoprolol–Ramipril −11.4%). This trend reverted at 12 months in all the drug arms (Ramipril −8.2%, Bisoprolol −9.4%, Bisoprolol–Ramipril −8.9%), whereas there was only a slight improvement in the Placebo arm (−12.2%). At 24 months, FAC showed a little improvement in the Placebo arm (−10.5%), whereas it further increased in all the drug arms almost returning to the baseline values (Ramipril −2.6%, *p* = 0.002, Bisoprolol −4.0%, Bisoprolol–Ramipril −4.2%, *p* < 0.001 for both). These changes were statistically significant (Greenhouse‐Geisser *F* = 2.637, *p* = 0.003).

Similarly, S'RV and TAPSE decreased in all the study arms at 3 and 6 months. S'RV continued to decrease at 12 months, except for the Bisoprolol arm, which showed an improvement. At 24 months, S'RV slightly improved in all patients, mainly in the intervention arms. The S'RV changes observed were statistically significant (Greenhouse‐Geisser *F* = 3.468, *p* < 0.001). TAPSE continued to decrease at 12 months, except in the Ramipril–Bisoprolol arm, which showed no changes at 12 months. At 24 months, all patients showed a little improvement in TAPSE, which was greater in the intervention arms. The TAPSE changes observed were statistically significant (repeated measures time*arm: Greenhouse‐Geisser *F* = 2.621, *p* = 0.003). Postoperative left‐side radiation therapy patients showed a lesser reduction in TAPSE (−4.2 ± 0.9% vs. −6.6 ± 0.7%, *p* = 0.032) and S'RV (−5.2 ± 0.8% vs. −8.3 ± 0.7%, *p* = 0.004) in comparison to the other patients at EOS.

At 3 months, the RV diameter increased by 4.2%, 3.5%, and 3.0% in the Placebo, Bisoprolol, and Ramipril–Bisoprolol arms, respectively. It increased by 1.7% in the Ramipril group, which was significantly less than placebo, *p* = 0.024. At 6 months, RV diameter increased further to 6.5% in Placebo and 6.3% in Bisoprolol, but only 4.5% in Ramipril–Bisoprolol and 2.8% in Ramipril (significantly less than placebo, *p* = 0.006). At 12 months, the RV diameter decreased in all drug arms (Bisoprolol 4.9%, Ramipril 2.1%, Ramipril–Bisoprolol 3.5%, all *p* < 0.001 vs. Placebo), whereas it continued to increase in Placebo (6.7%) and remained stable (6.8%) at 24 months. In the intervention arms, RV diameter almost returned to baseline values at 24 months (Ramipril 1.4%, Bisoprolol 2.1%, and Ramipril–Bisoprolol 2.2%, *p* < 0.001 for all vs. Placebo). These changes were statistically significant (Greenhouse‐Geisser *F* = 4.260, *p* < 0.001).

RAV increased in all arms at 3 months and then gradually decreased to baseline values at 24 months in the Placebo, showing a reduction from baseline in the intervention arms. However, these changes were nonsignificant (Greenhouse‐Geisser *F* = 0.634, *p* = 0.661).

SPAP increased progressively at 3, 6, and 12 months in Ramipril–Bisoprolol and Bisoprolol arms (11.6%, 11.7%, 14.1%, and 5.8%, 8.3%, 9.9%, respectively), and decreased to 7.7% and 5.4%, respectively at 24 months. In the Placebo and the Ramipril arm, SPAP increased less at 3, 6, and 12 months (3.5%, 6.0%, 7.6%, and 3.7%, 4.6%, 5.8%, respectively). At 24 months, it decreased in Ramipril (4.6%) and increased slightly in Placebo (9.2%). These changes were nonsignificant (Greenhouse‐Geisser *F* = 0.643, *p* = 0.647).

NT‐proBNP showed a progressive increase in all groups at 3, 6, and 12 months. By 24 months, it increased in the Placebo group, with no significant changes in the cardioprotective groups.

The overall RV CTRCD evaluated as a FAC decrease > 10% was 29.7%. It was statistically different in the arms: RV CTRCD was 49.2% in the Placebo arm, and 20.4%, 22.2%, and 23.3% in the Bisoprolol, Ramipril, and Ramipril–Bisoprolol arms, respectively (chi‐squared 16.093, *p* = 0.001).

**FIGURE 3:**
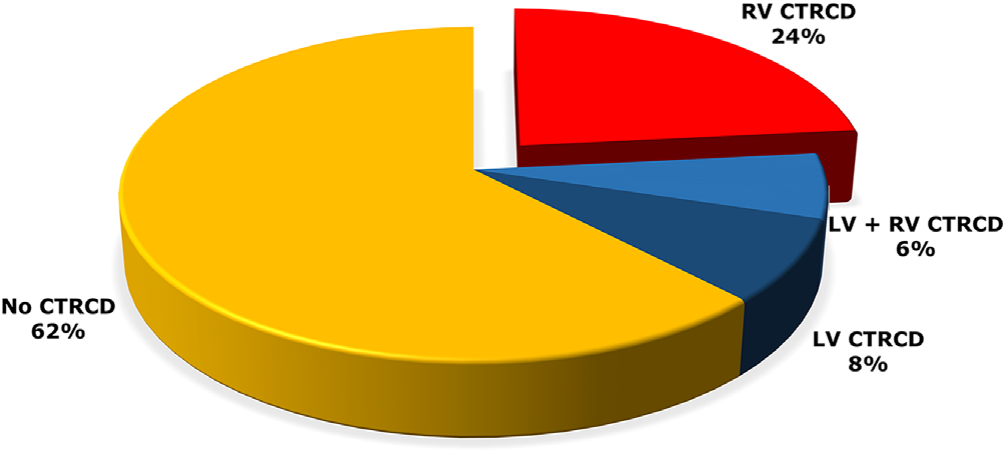
Pie chart of the distribution of cancer therapy–related cardiac dysfunction (CTRCD).

**FIGURE 4:**
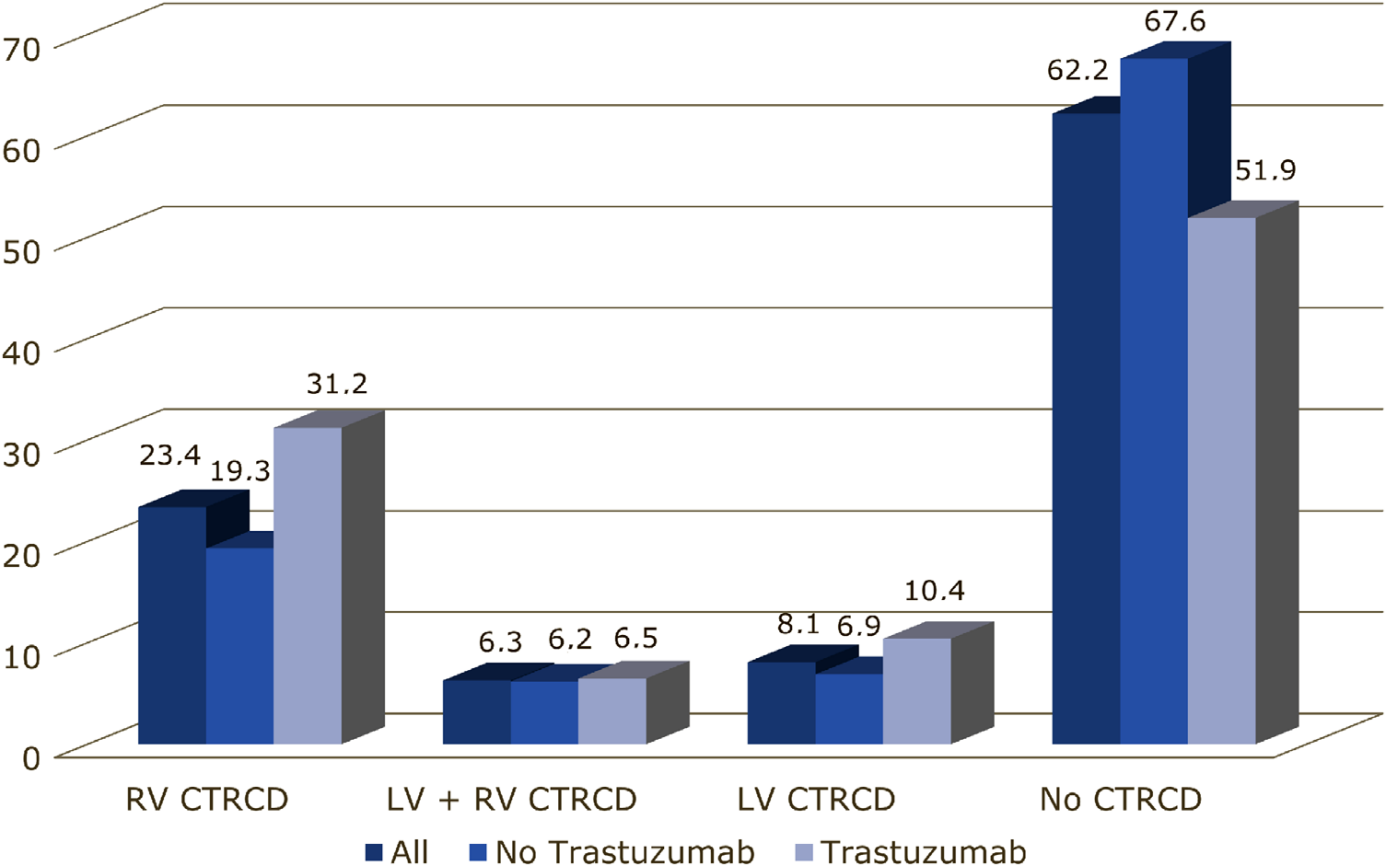
Histograms of the distribution and incidence of cancer therapy–related cardiac dysfunction (CTRCD) according to the treatment with trastuzumab or not. Bar color‐coding is reported under the graph. Abbreviations: CTRCD: cancer therapy–related cardiac dysfunction; LV: left ventricular; RV: right ventricular.

CTRCD involved mainly the right ventricle. It affected the right ventricle in 23.4% of patients, the left ventricle in 8.1%, and both ventricles in 6.3% of patients (Figure 3). CTRCD involving both ventricles was observed more frequently when trastuzumab was part of the chemotherapeutic regimen, although the difference was not statistically significant (Figure 4).

**FIGURE 5:**
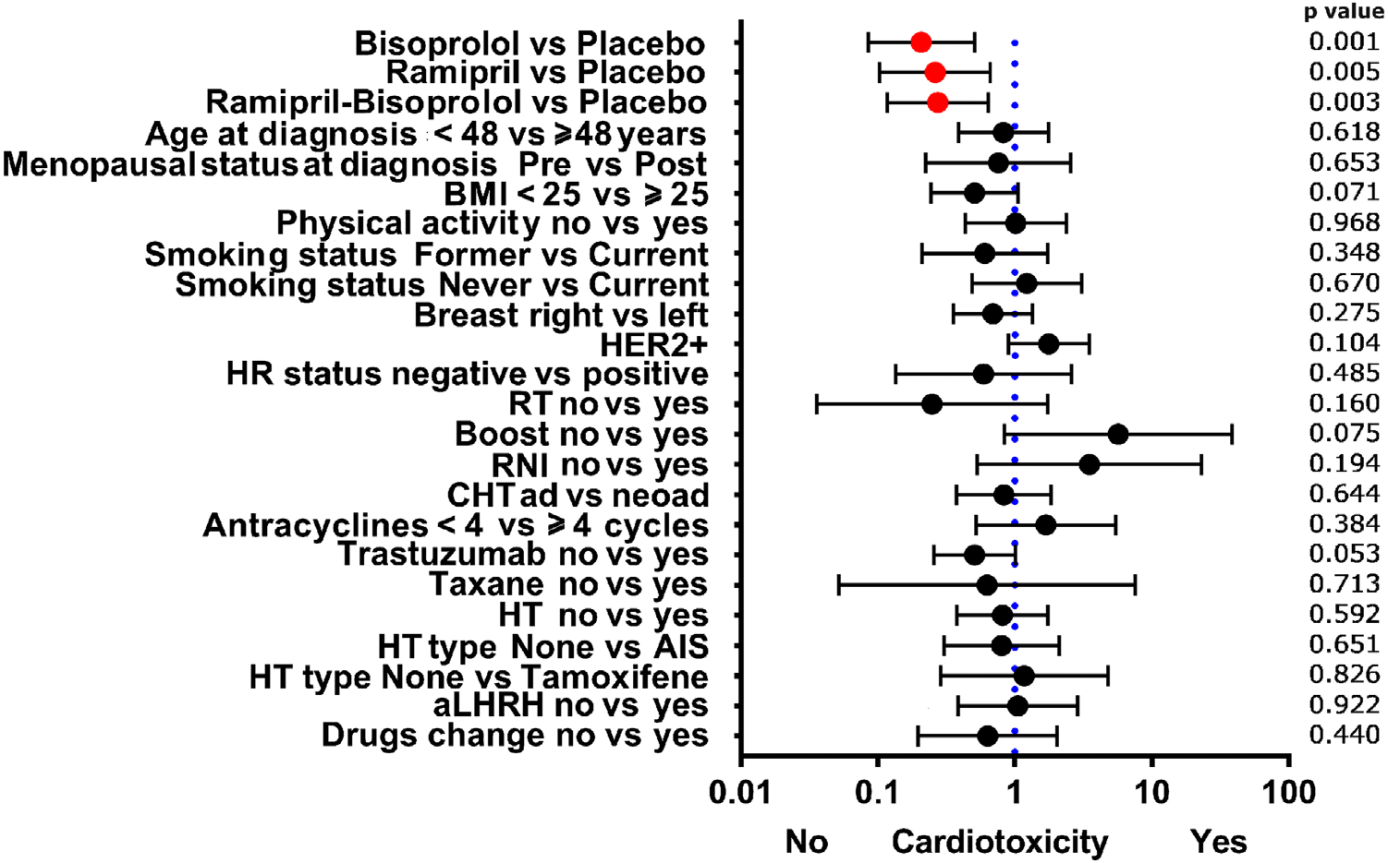
Forest plot of the determinants of right ventricular cardiotoxicity by univariate logistic analysis. In red, the drug arms versus the Placebo. Abbreviations: ad: adjuvant; AIS: aromatase inhibitors; aLHRH: luteinizing hormone‐releasing hormone agonist; BMI: body mass index; CHT: chemotherapy intent; HER2: human epidermal growth factor receptor 2; HR: hormonal receptor; HT: adjuvant endocrine therapy; neoad: neoadjuvant; RNI: regional nodal irradiation; RT: radiotherapy.

At univariate logistic analysis (Figure 5), the only factors significantly associated with the reduction in RV CTRCD were the treatment arms, with favorable outcomes compared to placebo for bisoprolol. Multivariate analysis (Table 3) showed a more favorable effect on RV damage compared to placebo for bisoprolol (odds ratio [OR] 0.235, 95% CI 0.100–0.551), ramipril (OR 0.273, 95% CI 0.113–0.660), and ramipril–bisoprolol (OR 0.305, 95% CI 0.137–0.676). Conversely, BMI > 25 (OR 2.067, 95% CI 1.079–3.963) and trastuzumab therapy (OR 1.932, 95% CI 1.027–3.634) had a small negative impact.

**TABLE 3:** Multivariate analysis.

Variable	Coefficient	Std Error	*p* value	Odds ratio	95% CI
Ramipril vs. Placebo	−1.2994	0.4512	0.004	0.273	0.113–0.660
Bisoprolol vs. Placebo	−1.4472	0.4345	0.001	0.235	0.100–0.551
Ramipril–Bisoprolol vs. Placebo	−1.1891	0.4070	0.003	0.305	0.137–0.676
BMI > 25	0.7263	0.3319	0.029	2.067	1.079–3.963
Trastuzumab	0.6584	0.3225	0.041	1.932	1.027–3.634
Constant	−0.4555				

*Note*: Statistical analysis: Full model ‐2 Log Likelihood = 245.513, Chi‐square = 24.687, *p* = 0.0002.

## 4 Discussion

The novelty of this study lies in the reduction of worsening of RV function in breast cancer patients who had a treatment with ACE inhibitors or β‐blockers or both, concomitant with the chemotherapy. Cardioprotection resulted in a lesser decline in RV function during the first 6 months, accompanied by a clear improvement in the following months. This was evident in the 12 months between the end of treatment and the end of follow‐up. In contrast, the placebo arm showed only mild improvement, alongside significant worsening compared to the intervention arms.

Currently, there is no recommendation for preventive treatment with ACE inhibitors, ARBs, or β‐blocker therapy in patients with low baseline cardiovascular risk receiving anthracyclines. Furthermore, no study has analyzed the protective effects of neurohormonal inhibitors on RV function. The present study addresses this gap by demonstrating that the therapy used to prevent LV dysfunction during chemotherapy can also help prevent chemotherapy‐related RV damage.

Although both the ACE inhibitor and the β‐blocker yielded the same long‐term results, the Ramipril arm showed a more favorable course during therapy. The reason for this remains unclear, but it may relate to the greater increase in pulmonary artery systolic pressure (PASP) observed in the Bisoprolol and Ramipril–Bisoprolol arms during the same period.

The study confirmed that anthracycline therapy adversely affects RV function. This cardiotoxic effect is evidenced by the simultaneous reduction of all parameters of RV function in the placebo arm. The worsening began in the third month, progressively increased up to 12 months, and remained unchanged in the following 12 months. This progression is in keeping with the knowledge that anthracycline damage is progressive.

Previous studies have shown that breast cancer patients receiving epirubicin or anthracycline‐based chemotherapy may experience a reduction in FAC or TAPSE [14, 15, 16]. However, there were no significant changes in PASP [15]. Furthermore, these studies included patients with hypertension, diabetes, and dyslipidemia [14, 17], and the effects were assessed at the end of chemotherapy [17, 18]. Notably, not all patients received anthracycline‐based chemotherapy [18].

In patients with initially normal RV function, a 10% decrease in FAC over several years resulted in RVD development in nearly a quarter of patients, which was strongly associated with negative outcomes [19]. The choice of a 10% FAC reduction, unlike the suggested decline below 35% [20] observed in only 17% of RV patients, better emphasizes the positive effects of cardioprotection.

Our data are similar to the results of Rossetto et al., who reported 28% RV CRTCD at 12 months [16] and Demissey et al., who showed an incidence of RV CTRCD of about 20% in the first 2 years [21].

The addition of trastuzumab to anthracyclines increased, although not significantly, the incidence of CTRCD in both the right and left ventricles. It was reported that the incidence of LV dysfunction and pathological abnormalities during trastuzumab therapy rose to 26%–28% when combined with anthracyclines [22, 23].

At EOS, patients who received left‐side radiation therapy showed a lesser worsening in TAPSE and S'RV than the other patients did. These data may differ from those published on the early effects of adjuvant radiotherapy [24]. However, the same research group reported a small reduction only in TAPSE, which did not differ significantly from the baseline at a 3‐year follow‐up [25].

The RV basal diameter in the placebo group exhibited a progressive increase during the first 6 months and then remained stable, suggesting mild compensatory remodeling. To the best of our knowledge, no such data are currently available in breast cancer patients treated with anthracyclines, apart from one study involving 96 patients diagnosed with diffuse large B‐cell lymphoma who received chemotherapy (cyclophosphamide, doxorubicin, vincristine, and prednisone plus rituximab) and were assessed using three‐dimensional transthoracic echocardiography [26]. However, further studies are necessary to understand the adaptive capacity of the right ventricle to damage.

CRTCD is not confined to the left ventricle, and the pathological changes are the same for the left and the right ventricles. Comparisons between LV CTRCD and RV CTRCD showed that isolated RV damage occurred in 23.4% of patients, suggesting that the right ventricle may be more vulnerable than the left ventricle to the toxic effects of chemotherapy due to its unique morphostructural features. The RV wall is thinner with fewer myofibrils than the thicker, more muscular LV walls. The free wall of the right ventricle primarily consists of subendocardial fibers oriented longitudinally, with the absence of mid‐layer circumferential fibers. Consequently, the subendocardial fibers, which are responsible for the majority of RV pump function, may be more susceptible to the toxic effects of chemotherapy. The time course of changes in TAPSE and S'RV supports this hypothesis.

The study has limitations. We lacked sensitive imaging measures, including RV global and free wall strain, although this is an area of future investigation. However, the parameters we describe are widely used and available, which enhances the generalizability of our findings, unlike RV strain. FAC, unlike TAPSE and TDI S velocity, reflects both RV free wall longitudinal strain and radial thickening. Additionally, FAC includes the contribution of the IVS in RV systolic assessment [27].

## 5 Conclusion

The most significant finding of our study is that short‐term neurohormonal cardioprotection reduced both LV CTRD and RV CTRCD in breast cancer patients undergoing anthracycline‐based chemotherapy. Cardioprotection with standard therapy for stage B cardiac dysfunction attenuated the decrease in RV function at 12 months compared to placebo, and allowed the recovery at 24 months with minimal loss of function. RV dysfunction persisted at 24 months in the placebo group. Logistic regression analysis indicates that only cardioprotection has a favorable effect on RV CTRCD, as opposed to clinical features, which have a neutral effect.

## Ethics Statement

The study protocol was approved by the institutional review board at each site and was conducted in accordance with the International Conference on Harmonisation Good Clinical Practice, the Declaration of Helsinki, and local regulations on the conduct of clinical research. All patients provided written informed consent before study participation.

## Conflicts of Interest

Icro Meattini reports personal fees from Eli Lilly, Novartis, Pfizer, Astra Zeneca, Daiichi Sankyo, Gilead, Menarini StemLine, outside the submitted work. Dr Olivotto reported grants from Myokardia, BMS, Shire, Takeda, Sanofi Genzyme, Amicus, and Bayer outside the submitted work. No other disclosures were reported.

## Data Availability

Lorenzo Livi and Giuseppe Barletta had full access to all the data in the study and took responsibility for the integrity of the data and accuracy of the data analysis. Qualified researchers may request access to individual patient‐level clinical data through a request to the principal investigator and after approval of the steering committee of the trial.
